# Systemic Down-Regulation of Delta-9 Desaturase Promotes Muscle Oxidative Metabolism and Accelerates Muscle Function Recovery following Nerve Injury

**DOI:** 10.1371/journal.pone.0064525

**Published:** 2013-06-13

**Authors:** Ghulam Hussain, Florent Schmitt, Alexandre Henriques, Thiebault Lequeu, Frederique Rene, Françoise Bindler, Sylvie Dirrig-Grosch, Hugues Oudart, Lavinia Palamiuc, Marie-Helene Metz-Boutigue, Luc Dupuis, Eric Marchioni, Jose-Luis Gonzalez De Aguilar, Jean-Philippe Loeffler

**Affiliations:** 1 INSERM, U1118, Mécanismes Centraux et Péripheriques de la Neurodégénérescence, Strasbourg, France; 2 Université de Strasbourg, UMRS1118, Strasbourg, France; 3 CNRS, UMR7178, Institut Pluridisciplinaire Hubert Curien, Equipe de Chimie Analytique des Molécules Bioactives, Illkirch, France; 4 CNRS, UPR9010, Institut Pluridisciplinaire Hubert Curien, Département d'Ecologie, Physiologie et Ethologie, Strasbourg, France; 5 INSERM, U977, Laboratoire des Biomatériaux et Ingéniérie Tissulaire, Strasbourg, France; University of Rome La Sapienza, Italy

## Abstract

The progressive deterioration of the neuromuscular axis is typically observed in degenerative conditions of the lower motor neurons, such as amyotrophic lateral sclerosis (ALS). Neurodegeneration in this disease is associated with systemic metabolic perturbations, including hypermetabolism and dyslipidemia. Our previous gene profiling studies on ALS muscle revealed down-regulation of delta-9 desaturase, or SCD1, which is the rate-limiting enzyme in the synthesis of monounsaturated fatty acids. Interestingly, knocking out SCD1 gene is known to induce hypermetabolism and stimulate fatty acid beta-oxidation. Here we investigated whether SCD1 deficiency can affect muscle function and its restoration in response to injury. The genetic ablation of SCD1 was not detrimental *per se* to muscle function. On the contrary, muscles in SCD1 knockout mice shifted toward a more oxidative metabolism, and enhanced the expression of synaptic genes. Repressing SCD1 expression or reducing SCD-dependent enzymatic activity accelerated the recovery of muscle function after inducing sciatic nerve crush. Overall, these findings provide evidence for a new role of SCD1 in modulating the restorative potential of skeletal muscles.

## Introduction

In mammals, the contraction of voluntary skeletal muscles is under the control of motor neurons whose cell bodies are located in the spinal cord and the brainstem. These so-called lower motor neurons directly communicate with muscle fibers through the neurotransmitter acetylcholine at the neuromuscular junctions. The progressive functional deterioration of this neuromuscular axis is typically found in neurodegenerative conditions such as amyotrophic lateral sclerosis (ALS). This disease, which is the most frequent adult-onset form of motor neuron disease, is characterised by motor neuron death, skeletal muscle atrophy and paralysis [1]. Studies conducted on genetic animal models of ALS showed that the process leading to motor neuron degeneration is not cell-autonomous but involves defects in other cell types than neurons [2]–[5]. Consistent with this notion, ALS neurodegeneration is also associated with systemic defects, including hypermetabolism and dyslipidemia, which are observed in both patients and animal models [6]–[8]. Particularly, transgenic mice overexpressing a mutated form of Cu/Zn superoxide dismutase (SOD1), linked to familial ALS, are in energy deficit and have decreased adipose tissue stores. These deficiencies appear to be elicited by increased energy expenditure (that is to say, hypermetabolism), and due to an increased consumption of nutrients by skeletal muscles [9], [10].

To gain insight into the relationships between neurodegeneration and metabolic dysfunction, we recently analyzed the gene expression profiles of skeletal muscles from mutant SOD1 mice and patients with sporadic ALS [11], [12]. We found a decrease in the expression of stearoyl-CoA desaturase-1 (SCD1), an enzyme that introduces the first *cis* double bond in the delta-9 position of saturated fatty acyl-CoA substrates. The preferred substrates are palmitoyl-CoA (C16:0) and stearoyl-CoA (C18:0), which are converted to palmitoleoyl-CoA (C16:1) and oleoyl-CoA (C18:1), respectively [13]. These monounsaturated fatty acids are the major constituents of complex lipids such as diacylglycerols, phospholipids, triglycerides, wax esters and cholesterol esters. Interestingly, the targeted disruption of the mouse SCD1 gene triggers an increase in the expression of genes involved in the β-oxidation of fatty acids and a decrease in the expression of genes involved in lipogenesis [14]. Therefore, these SCD1 knockout mice exhibit augmented energy expenditure and reduced body adiposity [14], a situation that is reminiscent of the metabolic phenotype of mutant SOD1 mice [9]. Despite sharing common features at the metabolic level, it is not known whether a decrease in SCD1 expression could be implicated by any means in the maintenance of muscle function and, perhaps, in motor neuron degeneration. In this work, first we characterized the expression of SCD1 in muscles from mutant SOD1 mice as well as in experimentally denervated muscles. Second, we studied the muscle phenotype of mice deficient in SCD1. Third, we analyzed the impact of the absence of SCD1, as obtained by both genetic and pharmacological means, on the recovery of muscle function in response to transient nerve lesion. We conclude that the systemic down-regulation of SCD1 promotes muscle oxidative metabolism and accelerates muscle function recovery after nerve injury, thus providing evidence for a new role of this enzyme in modulating the restorative potential of skeletal muscles. These findings therefore may be relevant to pathological conditions affecting the lower motor neurons.

## Materials and Methods

### Animals

FVB/N males overexpressing the murine G86R SOD1 mutation [15], and C57BL/6 males knockout for the SCD1 gene (The Jackson Laboratory, Bar Harbor, ME) were maintained in our animal facility at 23°C with a 12 hours light/dark cycle. They had water and regular A04 rodent chow *ad libitum*. SOD1(G86R) mice were 60-, 75-, 90- and 105 days of age. SCD1 knockout mice were 4–6 months old. The corresponding non-transgenic male littermates served as controls. To induce peripheral nerve injury, mice were anesthetized with 100 mg/kg body mass ketamine chlorhydrate and 5 mg/kg body mass xylazine. The sciatic nerve was exposed at the midthigh level, and crushed with a fine forceps for 30 s or sectioned 3 mm long with microscissors. Skin incision was sutured, and mice were allowed to recover. Hind limbs contralateral to the lesion served as controls [16]. To induce SCD deficiency pharmacologically, 4–6 months old mice were fed with A04 chow *ad libitum* containing 3-(5-methyl-[1], [3], [4]thiadiazol-2-yl)-6-[4-(2-trifluoromethyl-phenoxy)-piperidin-1-yl]-pyridazine, or MF-438 (Prestwick Chemical, Illkirch, France), which is an orally bioavailable inhibitor of SCD enzymatic activity [17]. The drug regimen was prepared to contain 0.00625% (w/w) MF-438 (Safe, Augy, France), which provided a daily dose of 10 mg/kg body mass, as calculated on a basis of 4 g of food intake per day and 25 g of averaged body mass.

### Ethics statement

Experiments followed current European Union regulations (Directive 2010/63/EU), and were performed by authorized investigators (license from *Prefecture du Bas-Rhin* No. A67-402 to AH, and No. A67-118 to FR), after approval by the ethics committee of the University of Strasbourg (license from CREMEAS No. AL/01/20/09/12, and No. AL/15/44/12/12).

### Quantitative RT-PCR

Total RNA was prepared following standard protocols. Briefly, each frozen sample was placed into a tube containing a 5-mm stainless steel bead. Working on ice, 1 mL Trizol reagent (Invitrogen, Groningen, The Netherlands) was added, and homogenisation was performed in a TissueLyser (Qiagen, Valencia, CA) at 30 Hz for 3 min twice. RNA was extracted with chloroform/isopropyl alcohol/ethanol and stored at –80°C until use. One μg of total RNA was used to synthesize cDNA using Iscript reverse transcriptase (BioRad Laboratories, Marnes La Coquette, France) and oligo-dT primer as specified by the manufacturer. Gene expression was measured using the SYBR green reagent (2× SYBR Green Supermix; Bio-Rad Laboratories) following the manufacturer's instructions on a Bio-Rad iCycler. PCR was performed in optimized conditions: 95°C denatured for 3 min, followed by 40 cycles of 10 s at 95°C and 30 s at 60°C. Primers (Eurogentec, Seraing, Belgium) were as follows: acetylcholine receptor α-subunit (AChR-α), forward 5′-ccacagactcaggggagaag-3′, reverse 5′-aacggtggtgtgtgttgatg-3′; acetylcholine receptor γ-subunit (AChR-γ), forward 5′-gagagccacctcgaagacac-3′, reverse 5′-gaccaacctcatctccctga-3′; acetylcholine receptor ε-subunit (AChR-ε), forward 5′-caatgccaatccagacactg-3′, reverse 5′-ccctgcttctcctgacactc-3′; muscle-specific receptor tyrosine kinase (MuSK), forward 5′-ttcagcgggactgagaaact-3′, reverse 5′-tgtcttccacgctcagaatg-3′; peroxisome proliferator-activated receptor α (PPARα), forward 5′-cacgcatgtgaaggctgtaa-3′, reverse 5′-gctccgatcacacttgtcg-3′; peroxisome proliferator-activated receptor γ, coactivator 1-α (PGC1-α), forward 5′-tgctgctgttcctgttttc-3′, reverse 5′-ccctgccattgttaagacc-3′; pyruvate dehydrogenase kinase, isoenzyme 4 (PDK4), forward 5′-cgcttagtgaacactccttcg-3′, reverse 5′-cttctgggctcttctcatgg-3′; ribosomal RNA 18S (18S), forward 5′-cgtctgccctatcaactttcg-3′, reverse 5′-ttccttggatgtggtagccg-3′; SCD1, forward 5′-cctacgacaagaacattcaatcc-3′, reverse 5′-cgtctcaagttctcttaatcct-3′. Relative quantification was achieved by calculating the ratio between the cycle number (Ct) at which the signal crossed a threshold set within the logarithmic phase of the gene of interest and that of the 18S reference gene. Ct values were the means of duplicates.

### Gas chromatography

Muscle samples were weighed, and disrupted with TissueLyser twice at 30 Hz for 3 min each using stainless steel beads and racks precooled at −80°C. After removing the beads, 1 mL ice-cold Ripa buffer was added per 100 mg of disrupted tissue, and samples were centrifuged at 2000 g for 10 min at 4°C. Two mg of proteins, according to the bicinchoninic acid assay in the supernatants, was mixed with 2.5 mL chloroform/methanol (1∶1). After vortex and sonication, samples were incubated for at least 2–3 hours at 4°C, and centrifuged at 2000 g for 10 min at 4°C. Supernatants were collected into glass tubes with a Pasteur pipette, and dried under nitrogen. Afterwards, the non-polar lipid fraction was obtained by separation on Sephadex columns. In the case of blood samples, they were collected on heparinized tubes by heart puncture from anesthetized mice, placed on ice, and centrifuged at 4000 rpm for 4 min at 4°C. Total lipids were extracted from plasma following a modified version of the Bligh and Dyer method [18]. Briefly, methanol/chloroform solution was added to plasma. Centrifugations were performed after addition of chloroform and KCl. Lower phases were collected and mixed with methanol. After a second centrifugation, the lower phase containing the lipids was collected on glass tubes. Extracted lipids from muscle and plasma were transmethylated by adding a mix of methanol and KOH. After collecting the samples in heptane, the so-generated fatty acid methyl esters were submitted to gas chromatography by using a Varian 3400 CX chromatograph fitted with a WCOT fused silica capillary column of 100 m×0.25 mm×0.20 µm (coated with polar highly substituted cyanopropyl CP-SIL 88 phase). The injection volume was 1 µL, and the split ratio was set at 1∶1. The temperature gradient in the oven ranged from 80 to 220°C at a rate of 4°C/min, and helium was the gas carrier. The temperature of the flame ionization detector was set at 270°C. Peaks were identified by retention time and compared to a standard mix of fatty acid methyl esters (Supelco 37 and Supelco PUFA-2 Animal Source; Sigma-Aldrich, Saint-Quentin Fallavier, France). Data were expressed as relative percentages.

### Muscle grip strength

Muscle strength was determined using the grip test (Bioseb, Chaville, France). Animals were placed over a metallic grid that they instinctively grab to try to stop the involuntary backward movement carried out by the manipulator until the pulling force overcomes their grip strength. After the animal loses its grip, the strength-meter scores the peak pull force. Strength was measured independently in hind limbs ipsi- and contralateral to the nerve lesion, and the mean of 3 assays was scored for each animal. Peak force was normalized to body mass.

### Muscle histochemistry

The standard histochemical assay for succinate dehydrogenase (SDH) was used to distinguish between oxidative and non-oxidative muscle fibers. Fourteen-μm serial sections were obtained by cutting isopentane fresh-frozen tibialis anterior muscles perpendicular to the muscle axis on a cryostat at –20°C (Leica, Nanterre, France). Sections were fixed with acetone for 15 min, air-dried and incubated for 30 min at 37°C in a solution containing 10 g/L sodium succinate, 2 g/L nitro blue tetrazolium and 0.025 g/L phenazine methosulfate. After washing, sections were mounted with aqueous mounting medium (Dako, Trappes, France). Microphotographs were obtained at a magnification of ×200 (Nikon, Champigny sur Marne, France) on five non-adjacent sections per animal. Counts were performed using National Institutes of Health IMAGE version 1.62 software.

### Indirect calorimetry

O_2_ consumption and CO_2_ production were measured using an open-circuit indirect calorimetry system (Klogor, Lannion, France), as previously described [9]. Concentrations of O_2_ and CO_2_ in the outgoing air were successively measured in five different cages. The system was rinsed for 90 s between each measurement. Final values of gas concentrations were the mean of 10 measures obtained during 40 s. Each cage was sampled every 11 min, and one cage was left vacant as reference of ambient gas concentrations. Measurements were performed continuously over 23 hours and a half, a 30-min period being required for calibration of the O_2_ and CO_2_ analyzers. In total, 127 measures were collected per day and mouse. The respiratory quotient was the ratio of CO_2_ production over O_2_ consumption.

### Electromyography

Recordings were obtained with a standard electromyography apparatus (Dantec, Les Ulis, France), in accordance with the guidelines of the American Association of Electrodiagnostic Medicine. Mice were anesthetized as indicated above and kept under a heating lamp to maintain a physiological muscle temperature (at about 31°C). A concentric needle electrode (no. 9013S0011, diameter 0.3 mm; Medtronic, Minneapolis, MN) was inserted in the gastrocnemius, and a monopolar needle electrode (no. 9013R0312, diameter 0.3 mm; Medtronic) was inserted into the tail of the mouse to ground the system. Each muscle was monitored in four different regions, and the degree of denervation was scored as the number of regions with spontaneous activity expressed in percentage. Spontaneous activity was differentiated from voluntary activity by visual and auditory inspection. Only spontaneous activity with a peak-to-peak amplitude of at least 50 μV was considered to be significant.

### Statistical analysis

Unless otherwise indicated, data are expressed as the mean ± SEM. PRISM version 5.0a software (GraphPad, San Diego, CA) was used for statistical analysis. Tests are indicated in the legends under the figures. Differences with *P*-values of at least less than 0.05 were considered significant.

## Results

### SCD1 expression is altered in ALS muscle

**Figure 1:**
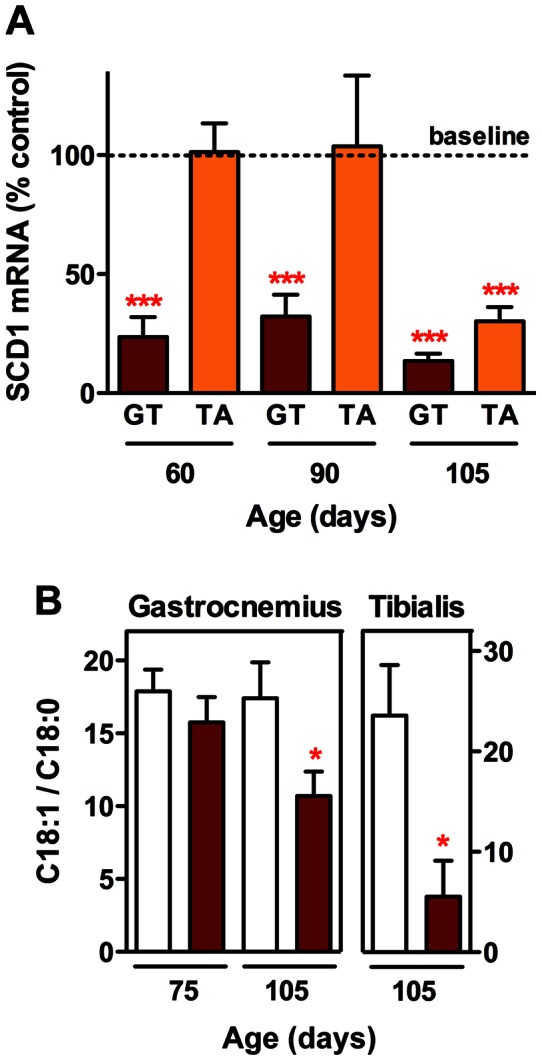
SCD1 expression and activity in ALS mouse muscle. (A) Time course of SCD1 expression in gastrocnemius (GT, brown columns) and tibialis anterior (TA, orange columns) from SOD1(G86R) mice at indicated ages. Wild-type expression is represented by 100% baseline. ***P<0.001 (One sample t-test, n = 5–11). (B) C18:1/C18:0 fatty acid ratio in gastrocnemius and tibialis anterior from SOD1(G86R) mice (brown columns) and wild-type littermates (white columns) at indicated ages. *P<0.05 (1-way ANOVA followed by Bonferroni's multiple comparison test for gastrocnemius, and unpaired *t*-test for tibialis anterior, n = 3–10).

On the basis of our previous microarray data, obtained from a transgenic mouse model of mutant SOD1-linked familial ALS [11], in this study we investigated the significance of the down-regulation of SCD1 for the metabolic capacity of muscles and their response to injury. The expression of SCD1 in the gastrocnemius of SOD1(G86R) mice, which are affected by a progressive denervation atrophy [19], was already diminished at 60 days of age. In this respect, it is noteworthy to mention that our previous electromyography studies on this mouse line revealed that the amplitudes of the compound muscle action potentials, a reduction of which typically reflects a decrease in the number of functional motor units, were normal at the age of 75 days. In addition, mice did not present at this age any abnormal spontaneous electrical activity, which would have reflected the common response of muscle to loss of innervation [16]. According to these findings, we can conclude that SCD1 down-regulation occurred precociously in our SOD1(G86R) mouse model. We then showed here that the decrease in SCD1 expression also persisted during the course of the disease, at 90 days of age, when muscle denervation becomes detectable and motor deficits usually arise, and at about 105 days of age, when hind legs start to be paralysed. At that moment, the decrease in SCD1 expression was also noticeable in the tibialis anterior, which is another muscle in the mouse hind leg displaying less oxidative metabolism than the gastrocnemius (Figure 1A). As a consequence of the repression of muscle SCD1 expression, we observed that the C18:1/C18:0 fatty acid ratio, an index of the desaturation activity of the enzyme [20], was slightly reduced in presymptomatic muscle extracts but significantly diminished at the end stage in both gastrocnemius and tibialis anterior (Figure 1B). It is noteworthy to mention that our previous studies had shown that SOD1(G86R) mice typically exhibit decreased postprandial lipidemia and increased peripheral clearance of lipids, both of which can be ascribed to muscle hypermetabolism [10]. Therefore, an excess of uptake of exogenous lipids in this tissue could mask otherwise earlier and more robust differences in the index of SCD activity.

**Figure 2:**
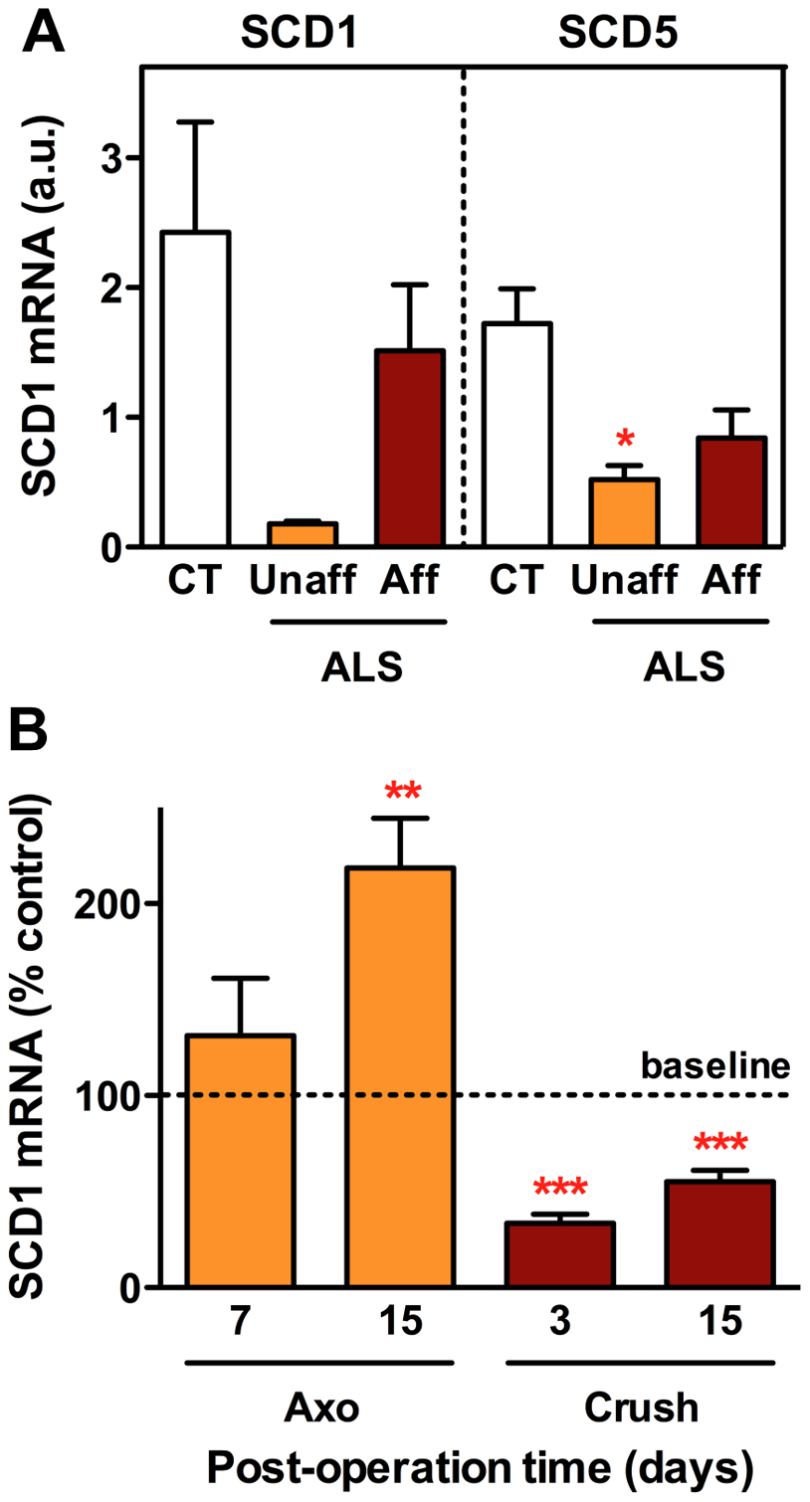
SCD1 expression in ALS patient muscle and after nerve injury. (A) Expression of SCD1 and SCD5 in deltoid muscle biopsies from ALS patients and healthy subjects (CT, white columns), as identified by microarray analysis of the database deposited at http://www.ebi.ac.uk/arrayexpress/(accession number E-MEXP-3260) [12]. ALS samples were obtained from muscle not clinically or electromyography affected (Unaff, orange columns) and from muscle with advanced pathology, characterized by reduced strength and neurogenic electromyography pattern (Aff, brown columns). *P<0.05 (1-way ANOVA followed by Tukey's multiple comparison test, n = 4–10). (B) Expression of SCD1 in gastrocnemius following sciatic nerve axotomy (Axo) or crush at indicated post-operation days. Contralateral muscle expression is represented by 100% baseline. **P<0.01, ***P<0.001 (One sample t-test, n = 4–10).

To obtain independent evidence that SCD1 down-regulation is a typical feature of ALS, we took advantage of our transcriptome database composed of deltoid biopsies from patients with the sporadic form of the disease [12]. The expression of not only SCD1 but also SCD5, a primate-specific enzyme variant with identical function [21], was lower in ALS patients, as compared to normal control subjects. Furthermore, the repression of SCD1 expression was much more remarkable in a muscle not clinically or electromyography affected than in a muscle at an advanced stage of pathology, characterized at the clinical level by reduced strength and neurogenic electromyography pattern (Figure 2A). That SCD1 down-regulation could be observed both in presymptomatic SOD1(G86R) mouse muscle and in relatively healthy human ALS muscle prompted us to speculate that such a pattern of expression might not be solely related to the loss of muscle innervation characteristic of the disease. To address this question, we compared SCD1 expression in gastrocnemius submitted to acute denervation, as obtained by cutting and removing several millimeters of the sciatic nerve, or transient denervation followed by re-innervation, as obtained by crushing the sciatic nerve for several seconds. Under these conditions, the expression of SCD1 was increased after axotomy but significantly reduced after crush (Figure 2B). Overall, these findings provide evidence for the implication of SCD1 in the pathological process triggering ALS, and suggest that SCD1 down-regulation could be involved in the restoration of muscle function in response to injury.

### SCD1 knockout mice do not manifest motor impairment but display exacerbated muscle metabolic oxidative capacity

**Figure 3:**
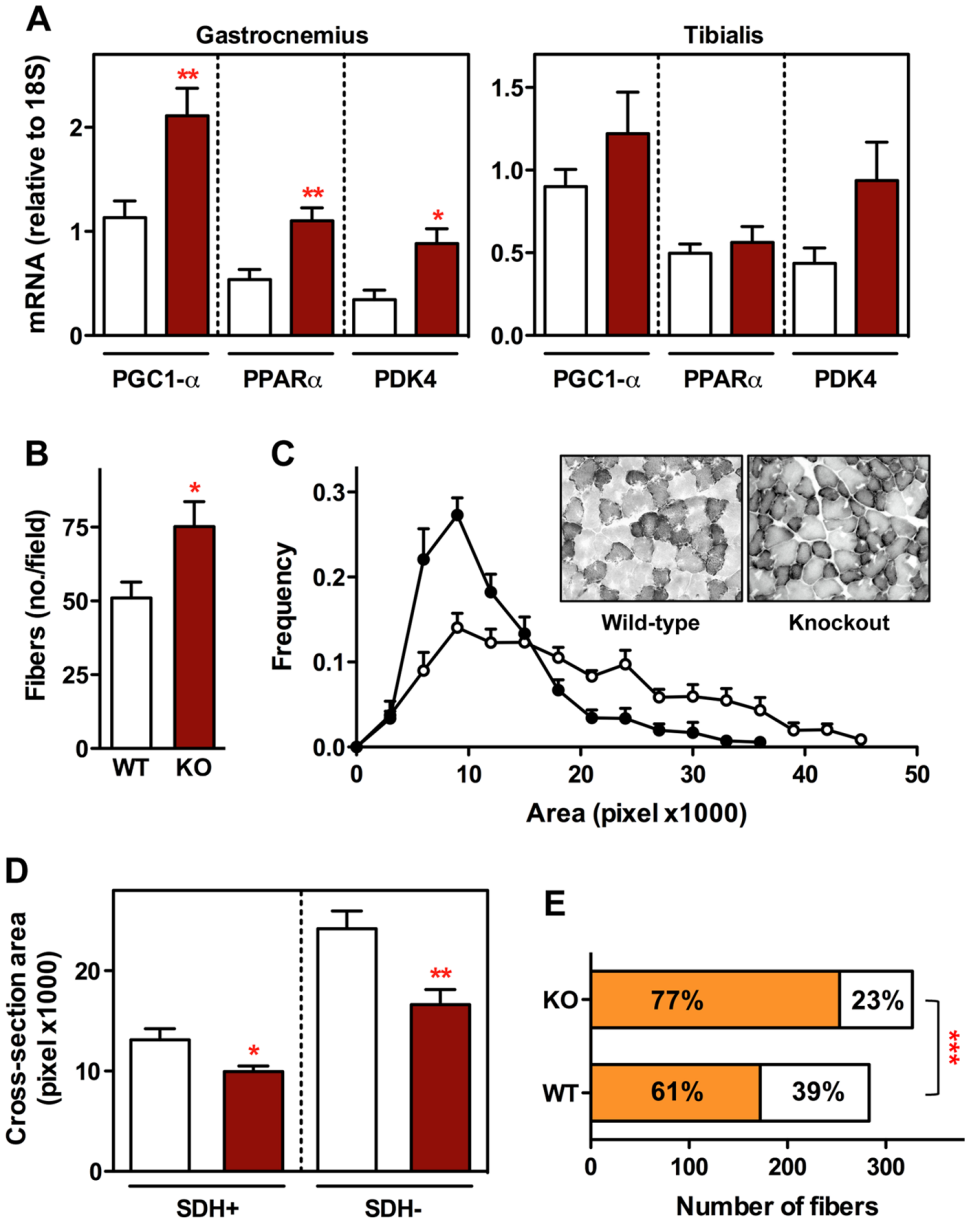
Metabolic phenotype of muscle from SCD1 knockout mice. (A) Expression of PGC1-α, PPARα and PDK4 in gastrocnemius and tibialis anterior from SCD1 knockout mice (brown columns) and wild-type littermates (white columns). *P<0.05, **P<0.01 (Unpaired t-test, n = 3–11). (B) Number of muscle fibers in tibialis anterior from SCD1 knockout mice (KO, brown column) and wild-type littermates (WT, white column). *P<0.05 (Unpaired t-test, n = 7–10). (C) Distribution of the calibers of muscle fibers in tibialis anterior from SCD1 knockout mice (327 fibers, black circles) and wild-type littermates (283 fibers, white circles). Representative microphotographs of wild-type and knockout tibialis anterior are shown. (D) Averaged cross-sectional area of SDH-positive and SDH-negative fibers in tibialis anterior from SCD1 knockout mice (brown columns) and wild-type littermates (white columns). *P<0.05, **P<0.01 (Unpaired t-test, n = 7–10). (E) Number of SDH-positive (orange bars) and SDH-negative fibers (white bars) in tibialis anterior from SCD1 knockout mice (KO) and wild-type littermates (WT). ***P<0.001 (Chi-square test, n = 283–327).

To gain insight into the way in which the lack of SCD1 expression impacts on muscle function, we investigated several characteristics of muscles in these SCD1 knockout mice reflecting their metabolic status, and also evaluated their motor behavior. At the molecular level, we measured the expression of PGC1-α, PPARα and PDK4, of which an increase is involved in stimulating mitochondrial biogenesis and in switching the energy source from glucose to fatty acids [22]. The expression of these genes was significantly higher in the gastrocnemius of SCD1 knockout mice, as compared to wild-type littermates; in the tibialis anterior, there was also a trend toward an increased expression (Figure 3A). Despite this latter attenuated response, the tibialis anterior represents, better than the gastrocnemius, a typical example of glycolytic muscle in which to evaluate changes in the relative density of the various fiber types. We therefore used this muscle to determine potential morphological and biochemical changes triggered by the absence of SCD1. The number of fibers per muscle section was higher in SCD1 knockout mice than in wild-type mice (Figure 3B). Accordingly, the distribution of fiber calibers showed an increase in the amount of fibers of small caliber in SCD1 knockout mice (Figure 3C). We extended these findings by performing SDH histochemistry, and found that the average cross-sectional area of both SDH-positive and SDH-negative fibers was smaller in SCD1 knockout mice than in wild-type mice (Figure 3D). These differences were associated in SCD1 knockout mice with a significant predominance of SDH-positive fibers, which are characterized by a higher metabolic oxidative capacity (Figure 3E).

**Figure 4:**
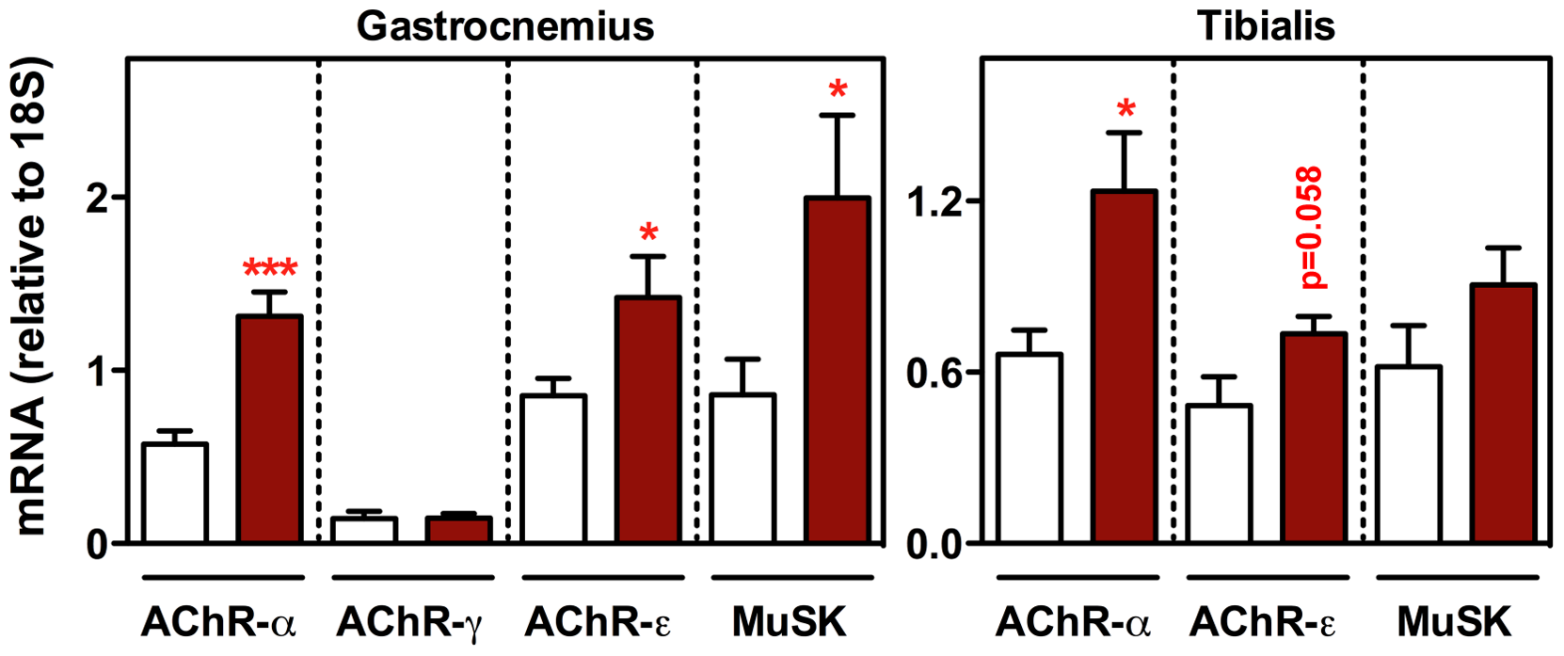
Gene expression specific to the motor end plate in SCD1 knockout mice. Expression of AChR-α, AChR-γ, AChR-ε and MuSK in gastrocnemius and tibialis anterior from SCD1 knockout mice (brown columns) and wild-type littermates (white columns). *P<0.05, ***P<0.001 (Unpaired t-test, n = 4–11).

Evaluation of muscle function using the grip strength test revealed no changes in the force developed by hind limbs between SCD1 knockout mice and their wild-type littermates (0.37±0.024 N in SCD1 knockout mice *versus* 0.37±0.021 N in wild-type mice, n = 7). Along with this, no abnormal spontaneous electrical activity, which would have reflected the typical response of muscle to loss of innervation, was found in the gastrocnemius of SCD1 knockout mice (data not shown). In contrast, we also measured the expression of a series of genes specific to the motor end plate, including the acetylcholine receptor subunits α, γ and ε (AChR-α, AChR-γ and AChR-ε, respectively), and muscle-specific receptor tyrosine kinase (MuSK). Except for AChR-γ, of which an increase would have been considered a sign of muscle denervation [23], the expression of these genes was significantly increased in the gastrocnemius of SCD1 knockout mice as compared to their wild-type littermates, although the changes were less pronounced in the tibialis anterior (Figure 4). In all, these results indicate that the genetic ablation of SCD1 is not detrimental *per se* to muscle function but promotes a metabolic shift toward a more oxidative capacity, and stimulates the neuromuscular junction gene expression program.

### SCD deficiency accelerates muscle function recovery after nerve injury

**Figure 5:**
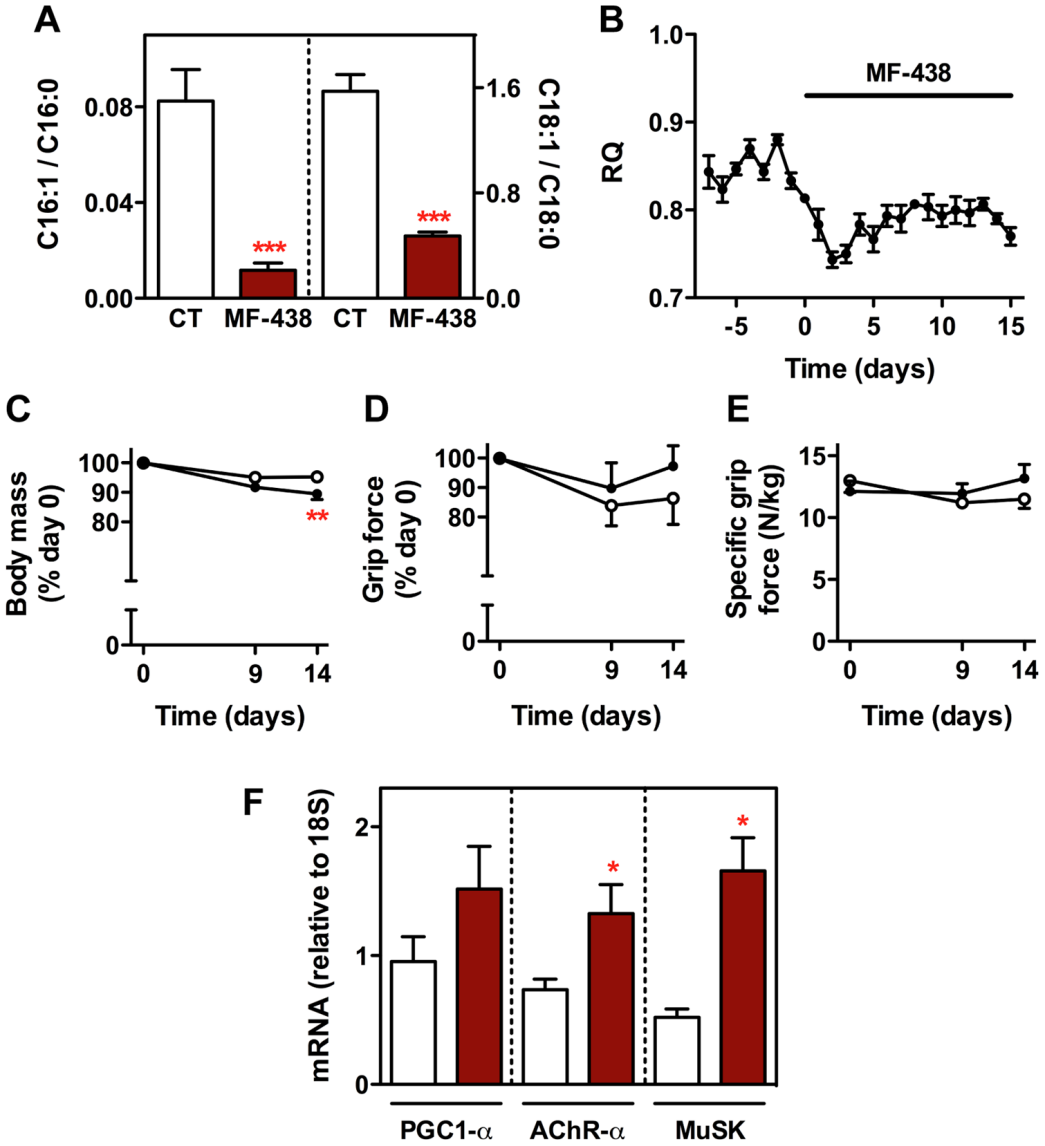
Effects of MF-438 on metabolism and muscle function. (A) C16:1/C16:0 and C18:1/C18:0 fatty acid ratio in plasma from MF-438 treated mice (brown columns) and control littermates (CT, white columns). ***P<0.001 (Unpaired t-test, n = 4–6). (B) Time course of respiratory quotient (RQ) before and after treatment with MF-438 at a dose of 10 mg/kg body mass/day (indicated by the black bar) (n = 4). Time course of body mass (C), muscle grip strength expressed as a percentage of day 0 (D), and specific grip strength, as determined by normalizing peak force to body mass (E), in mice fed regular chow (white circles) and mice fed regular chow supplemented with MF-438 (black circles). **P<0.01 (2-way ANOVA followed by Bonferroni test, n = 5–6). (F) Expression of PGC1-α, AChR-α, and MuSK in gastrocnemius from MF-438 treated mice (brown columns) and control littermates (white columns). *P<0.05 (Unpaired t-test, n = 3–9).

As shown above, the lack of SCD1 expression does not represent a handicap for muscle function. Therefore, its down-regulation, as observed in ALS or after nerve crush, prompts us to hypothesize that the enzyme may participate in the restorative efforts that muscles experience at the early stages of disease, when neuromuscular deterioration is not generalized yet, or during the process of recovery following a brief disruption of the neuromuscular communication. To address this question, we took advantage of such a model of transient denervation and re-innervation as a means to evaluate, by performing relatively manageable short-term experiments [3], the importance of SCD1 for the restoration of muscle function in response to nerve damage. We performed these experiments using not only SCD1 knockout mice but also mice deficient in SCD enzymatic activity, as obtained by feeding them with MF-438, which is an orally bioavailable pharmacological agent inhibiting SCD-dependent desaturation of fatty acids [17]. To verify if our treatment was biologically active *in vivo*, we measured several parameters that should reflect the deficiency in SCD enzymatic activity. First, MF-438 significantly reduced both C16:1/C16:0 and C18:1/C18:0 fatty acid ratios in circulating lipids (Figure 5A). Second, MF-438 also induced a decrease in the respiratory quotient as determined by indirect calorimetry, which indicated a switch of the energy source from glucose to fatty acids (Figure 5B). Finally, the drug triggered concomitantly a small but significant decrease in body mass of mice treated for two weeks (Figure 5C). SCD deficiency did not alter hind limb grip strength during the 2-week treatment, as determined either by a percentage of peak force at day 0 (Figure 5D) or by normalizing peak force to body mass (Figure 5E). Also, there were no detectable electromyography abnormalities suggestive of denervation (data not shown). Furthermore, MF-438 stimulated the expression of several genes specific to the motor end plate (Figure 5F). Overall, these findings strongly support that the effects of the SCD inhibitor are very similar to those observed in SCD1 knockout mice.

**Figure 6:**
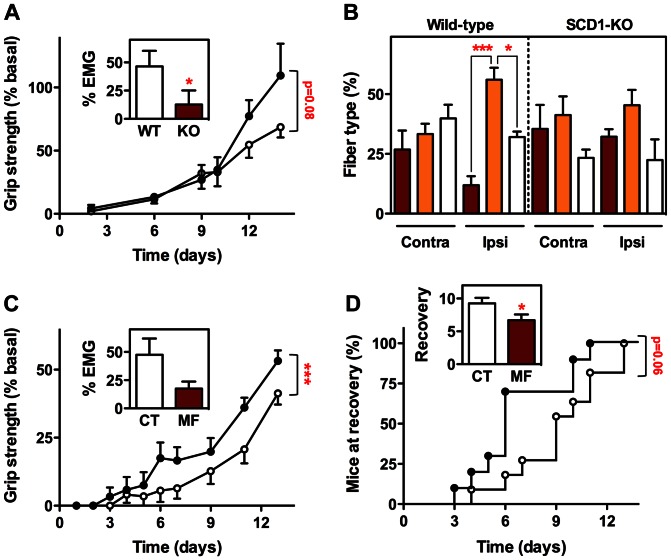
Muscle function recovery in SCD-deficient mice submitted to nerve crush. Restoration of muscle grip strength in SCD1 knockout mice (A) or MF-438 treated mice (C) (black circles) and corresponding control littermates (white circles) at the indicated post-operation times. ***P<0.001 (2-way ANOVA, n = 4–12). Percentage of electromyography episodes of spontaneous activity in SCD1 knockout mice (Inset A) or MF-438 treated mice (Inset C) (KO or MF, brown columns) and corresponding control littermates (WT or CT, white columns) two weeks after sciatic nerve crush. *P<0.05 (Unpaired t-test, n = 4–7). (B) Relative density of muscle fiber types in ipsilateral and contralateral tibialis anterior from SCD1 knockout mice and wild-type littermates two weeks after sciatic nerve crush. According to SDH histochemistry, fibers were classified as dark brown colored fibers with high metabolic oxidative capacity (brown columns), pale brown colored fibers with medium oxidative capacity (orange columns) and non-satined fibers (white columns). *P<0.05 and ***P<0.001 (1-way ANOVA followed by Tukey's multiple comparison test, (n = 4–6). (D) Kaplan-Meier curves showing the percentage of MF-438 treated mice (black circles) and control littermates (white circles) that started to exhibit a grip strength distinct from zero after initial total paralysis. Logrank test (n = 10–11). Inset D, averaged time at start of recovery in MF-438 treated mice (MF, brown column) and control littermates (CT, white column). *P<0.05 (Unpaired t-test, n = 10–11).

To monitor the recovery of muscle function after crushing the sciatic nerve, we measured hind limb grip strength during a post-lesion period of two weeks, and found that the force in SCD1 knockout mice was restored to its initial level more rapidly than in their wild-type littermates. Accordingly, the proportion of abnormal electromyography episodes reflecting neurogenic muscle denervation was lower in SCD1 knockout mice at 14 days post-lesion (Figure 6A). We also measured at that time the relative density of muscle fiber types as a witness to the restorative process. In the denervated tibialis anterior of wild-type mice, the proportion of fibers intensely stained by SDH histochemistry (presumably, slow-twitch type I fibers) was very low, and there was a significant predominance of medium-stained fibers (presumed fast-twitch fatigue-resistant type IIA fibers). In contrast, the distribution of fiber types in the denervated tibialis anterior of SCD1 knockout mice was identical to that observed in the muscle contralateral to the lesion (Figure 6B), suggesting the establishment of a normal non-stressed situation. To corroborate these findings, we followed muscle function recovery after crush in MF-438 treated mice, and also found accelerated, though not complete, restoration of grip strength, as well as reduced extent of electromyography abnormalities (Figure 6C). Furthermore, quantification of the percentage of mice that, after initial total paralysis, started to exhibit a grip strength distinct from zero showed that on average the recovery in treated mice took place three days sooner than in untreated mice (Figure 6D). In all, these results strongly suggest that reducing SCD enzymatic activity stimulates the restorative potential of skeletal muscles.

## Discussion

SCD1 is an essential lipogenic enzyme thought to be implicated in the development of obesity and associated metabolic disorders [24]. In this study, we have shown that its expression is down-regulated in skeletal muscles suffering from slowly progressing motor neuron degeneration, as seen in ALS, and from transitory denervation/re-innervation, as obtained experimentally by sciatic nerve crush. Based on these findings, we have also shown here that the systemic down-regulation of SCD1, as generated by both genetic and pharmacological means, enhances the oxidative metabolism of muscles, stimulates the expression of synaptic genes, and ameliorates the restoration of muscle function following transient denervation and subsequent re-innervation.

SCD1 knockout mice are mainly characterized by decreased adiposity and increased metabolic rate [14], two characteristics of the mutant SOD1 mouse model of ALS, even observed before the onset of any motor neuropathology [9]. The lack of SCD1 expression has been associated with inhibition of lipogenesis and stimulation of mitochondrial β-oxidation of fatty acids [25]. Notably, we had previously observed that the expression of genes involved in the uptake of fatty acids, such as FAT/CD36 and other related genes, was presymptomatically increased in skeletal muscle of SOD1(G86R) mice [10]. In addition, it is known that the muscle-specific overexpression of FAT/CD36 is sufficient to trigger fatty acid oxidation [26]. It seems therefore not very surprising that, in accordance with an enhanced uptake of fatty acids, the muscles of SOD1(G86R) mice exhibit concomitantly a decrease in the expression of SCD1. The situation appears to be less straight-forward in the case of ALS patients. Although most of them are markedly hypermetabolic [6], as in the animal model, they usually present with increased levels of circulating lipids [7], which would not intuitively substantiate the hypothesis of the muscle down-regulation of SCD1. It is noteworthy, however, that the expression of SCD1 in patients was much more repressed in muscles not clinically or electromyography affected, which lets envisage that SCD1 down-regulation might occur as a result of whatever mechanism preceding overt disease. The fact that SCD1 down-regulation was observed early in presymptomatic SOD1(G86R) mouse gastrocnemius and in relatively healthy human ALS deltoid suggests that the transcriptional regulation of SCD1 might not be (solely) related to the loss of innervation of muscles characteristic of these conditions. In support of this notion, such a down-regulation of SCD1 expression was not found after severe denervation, at least at a time when re-innervation was not present according to our protocol of axotomy. In contrast, a more subtle denervation, as that obtained by crushing the sciatic nerve for only several seconds, simulated the inhibitory effect of ALS on SCD1 expression. Because sciatic nerve crush allows the rapid recovery (in around two weeks) of muscle function, we speculate that SCD1 down-regulation may be somehow related to the restorative potential of skeletal muscles.

As a first step toward the understanding of the importance of SCD1 for muscle function, we found that the genetic ablation of SCD1 promoted a decrease in the cross-sectional area of muscle fibers, and an increase in the amount of those enriched in the mitochondrial enzyme SDH. In parallel, we also observed increased expression of PGC1-α, PPARα and PDK4. Overall, these observations provide persuasive evidence for a higher metabolic oxidative capacity, in accordance with several previous studies. For instance, overexpressing PGC1-α has been reported to trigger a decrease in the size of muscle fibers and a concomitant fast to slow fiber type shift [27]. In contrast, knocking out PGC1-α specifically in muscle has been shown to induce a shift from oxidative to glycolytic muscle fibers [28]. Moreover, PGC1-α not only triggers metabolic changes in muscle but also activates the expression of genes specific to the motor end plate [29]. Therefore, the increased expression of the signaling kinase MuSK and several acetylcholine receptor subunits that we observed in our SCD1 knockout mice could be the consequence of a higher transcriptional activity involving, at least in part, PGC1-α. Despite these modifications, the lack of SCD1 did not cause any alteration of muscle function as determined by behavioral and electrophysiological means. Furthermore, inhibiting global SCD enzymatic activity with a diet containing MF-438 induced very similar changes as those found in SCD1 knockout mice.

Subsequent efforts were concentrated in determining if SCD1 down-regulation might affect muscle function when challenged by sciatic nerve crush. Both SCD1 knockout mice and MF-438 treated mice recovered their force more rapidly than their corresponding control groups. Two weeks after lesion, the degree of denervation was more important in these control groups than in SCD-deficient mice. In support of these findings, very early studies had already reported that oxidative muscles can recuperate better than glycolytic ones after nerve crush [30]. Also, PGC1-α has been shown to protect muscles from denervation atrophy [31]. It can be therefore postulated that the pro-restorative power of SCD deficiency is the result of an enhancement of oxidative metabolism, as that shown in this work. A fast to slow fiber type shift has been shown to occur progressively in ALS, and it has been proposed that this metabolic modification would render muscle fibers with higher oxidative capacity more resistant to disease [32]. Recent studies have further reported that a modest increase in the expression of MuSK, as that observed here, can maintain neuromuscular junctions in mutant SOD1 mice, hence retarding denervation and ameliorating muscle function [33]. Taking our present findings as a whole, we can put forward that SCD1 stands at a regulatory crossroad shaping muscle function in health and disease. In this respect, the beneficial effects of inhibitors of SCD enzymatic activity could pave the way for developing novel therapeutic strategies to palliate motor neuron injury.
